# Effect of diode laser irradiation on bond strength of resin cements to high-translucent zirconia

**DOI:** 10.4317/jced.61953

**Published:** 2024-10-01

**Authors:** Giselly Cristina Matei, Daiane Cristina Peruzzo, Kamila Rosamilia Kantovitz, Aguinaldo Silva Garcez Segundo, Leonardo Santos Barros, Fabiana Mantovani Gomes França

**Affiliations:** 1 DDS, MSc, São Leopoldo Mandic Dental School, São Leopoldo Mandic Research Institute, Campinas, SP, Brazil; 2 DDS, MSc, PhD, São Leopoldo Mandic Dental School, São Leopoldo Mandic Research Institute, Campinas, SP, Brazil

## Abstract

**Background:**

Treatment of acid-resistance high-translucent zirconia prior to luting procedures is usually carried out with sandblasting. Considering that this process could lead to the formation of cracks on the zirconia surface, laser irradiation became an alternative to sandblasting. The effect of diode laser, however, was never investigated under this context. Therefore, this study aimed to evaluate the effect diode laser irradiation on the bond strength between zirconia and two resin cements after 48-hour and 12-month water aging.

**Material and Methods:**

Slabs of high-translucent zirconia were sandblasted with 50-µm Al2O3 particles (SB), irradiated with diode laser (DL), or SB+DL. All slabs were etched with 10% hydrofluoric acid and layered with a universal adhesive. Plastic tubes with 0.8-mm internal diameter positioned on treated zirconia surfaces were used as matrices to insert a dual- (DUAL) or a light-cure (LC) resin cement. All specimens were light-cured for 40 seconds at 1000 mW/cm2 and submitted to the µSBS test after 48-hour or 12-month water storage. Data were statistically analyzed with a three-way analysis of variance (*p*<0.05).

**Results:**

The µSBS of DUAL was higher than LC only after 12 months for SB or SB+DL-treated zirconia (*p*<0.05); in addition, SB+DL resulted in higher µSBS than DL only for DUAL resin cement (*p*<0.05). The 12-month aging resulted in higher µSBS for DUAL resin cement luted on SB or SB+DL-treated zirconia, as well as for LC luted on DL-treated zirconia (*p*<0.05). Most of the DUAL specimens presented adhesive failures after 48 hours. After 12 months, the majority of DUAL specimens luted on DL or SB+DL-treated zirconia presented adhesive failures.

**Conclusions:**

The use of dual-cure resin cement on sandblasted or sandblasted and diode laser-irradiated zirconia exhibited superior bond strength after aging.

** Key words:**Microshear bond strength, Resin cement, Diode laser, Zirconia, Sandblasting.

## Introduction

Dental ceramics are highly indicated indirect materials in oral rehabilitation due to their elevated mechanical resistance, biostability and optical properties (1). Among diverse types of ceramics, zirconia has been widely used considering its high biocompatibility and strength (2). Still, its use was limited to the posterior region, since the absence of a glass matrix in its composition resulted in less translucency, in comparison to silica-based ceramics (2). Developments over the past years led to the addition of 5 mol% yttria to the composition of zirconia, stabilizing it and maintaining the cubic phase content, thus resulting in a high-translucent zirconia, which allowed its use for anterior rehabilitation (3). Moreover, yttrium oxides also increase mechanical strength and reduce the aging degradation of zirconia (4-7).

In addition to the appropriate selection of composite materials, the adhesive luting of zirconia restorations to tooth structure is also crucial to the success of indirect restorations. It requires the surface treatment of both surfaces prior to the luting procedure itself (3,8,9). Sandblasting with aluminum oxide (Al2O3) particles is a surface treatment strategy that produces a micro-roughened zirconia surface that enhances adhesive bonding due to increased surface energy and wettability (9); however, sandblasting with inadequate Al2O3 particle size, excessive pressure, and/or time can create superficial microcracks and compromise the long-term mechanical strength of zirconia (10). Therefore, some authors suggest that the sandblasting protocol with 50-µm Al2O3 particles at 0.2 MPa pressure from a distance of 10 mm for 10 seconds is suitable for high-translucent zirconia surface treatment (7).

Even so, there are methods that can be used as an alternative to sandblasting or can even be used in combination with it. An example is the use of lasers, whose high amount of energy accumulated by them is transformed into heat that can be concentrated on a target area and modify the surface under controlled conditions (11). Initiation of ablation depends on absorption mechanisms, particle characteristics, infrastructures, and morphology; thus, each laser type has diverse reactions on different surfaces (9,10). Er,Cr:YSGG, Nd:YAG and FEMTO laser irradiation are examples of surface treatment of zirconia reported in the literature (12,13). The high-power Nd:YAG laser with a wavelength of 1064 nm, for instance, increases the surface roughness of zirconia and fuses the most superficial layer into a smooth and blister-like surface (11). Its use as treatment of zirconia surface has already been reported to result satisfactory bond strength to resin cements (14). However, it has been indicated that the influence of cracks originated after laser irradiation in the mechanical properties of zirconia could compromise the bond stability of zirconia-luting agent interface (15).

Diode laser has been used for diverse purposes in Dentistry, such as peri-implantitis treatment and gingivectomy (16,17). However, the use of diode laser irradiation as surface treatment of ceramics prior to luting procedures has not yet been investigated. Stübinger *et al*. (18) showed that diode laser was the best system in providing surface preservation and safety when used for treatment of yttria-stabilized tetragonal zirconia polycrystal (Y-TZP) implants. Considering that the aim of laser irradiation of zirconia restorations prior to the luting procedure is the formation of a rough surface, but with structural preservation and without cracks that could jeopardize bond stability, diode laser could be an interesting choice when irradiated at a similar wavelength (970 nm) to Nd:YAG laser.

The combination of a surface treatment that increases the micromechanical retention of high-translucent zirconia and a composite luting agent that provides strong chemical bonding is mandatory to establish long-term durable bond strength (8). Dual-cure resin cements are the most indicated to lute indirect restorations since their chemical cure ensures homogeneous and continuous monomer conversion at deep areas without light irradiation; thus, their enhanced mechanical properties are maintained in the long term (19,20). Considering that high-translucent zirconia can be used for anterior restorations, such as ceramic veneers, the choice of a resin cement that does not compromise the esthetic outcome becomes necessary. In this context, light-cure resin cements are indicated for this case as they do not possess tertiary amine in their composition, which could lead to oxidation and discoloration of the cementing line (21). Still, light-cure luting agents do not possess the same conversion degree and, consequently, properties as dual-cure. Therefore dual-cure resin cements without tertiary amine were developed, maintaining the properties of a dual-cure resin cement and improving their color stability (21).

Based on this rationale, this study aimed to evaluate the effect of different surface treatments and resin cement types on the bond strength to high-translucent zirconia after 48 hours and 12 months. Specifically, whether the diode laser could be used in combination or as an alternative method to sandblasting. The null hypotheses were that the surface treatment and resin cement type did not have a significant effect on the microshear bond strength (µSBS) to CAD/CAM zirconia blocks.

## Material and Methods

-Study design

This study had three independent variables following a 3 x 2 x 2 factorial scheme, as follows:

• Surface treatment: 1) SB = sandblasting with 50-µm Al2O3, particles; 2) DL = irradiation with diode laser; and 3) SB+DL.

• Resin cement: 1) DUAL = dual-cure resin cement (Rely X Ultimate, 3M ESPE, St. Paul, MN, USA); and 2) LC = light-cure resin cement (Rely X Veneer, 3M ESPE, St. Paul, MN, USA).

• Storage time: water storage for 1) 48 hours; and 2) 12 moths.

The experimental units comprised sixty slabs of high-translucent zirconia distributed in 6 groups (n = 10) resulted in the combination of surface treatments and resin cements.

-Sample preparation

All materials used in this study are described in  Table 1. Sixty slabs (10 x 10 x 5 mm) were milled from high-translucent zirconia CAD/CAM blocks (Ceramill Zolid FX Multilayer 71M, AmannGirrbach) and sintered for 2 hours up to 1450°C. Then, zirconia slabs were submitted to surface treatment according to the group to which they were randomly assigned. SB group were sandblasted with 50-µm Al2O3, particles from a distance of 40 mm during 20 seconds with 30-psi pressure and then cleaning through a 3-min ultrasonic bath. DL group was irradiated with 2W-power diode laser (Einstein DL, DC International, Carmel, IN, USA) for 25 seconds at 20 Hz with burst energy of 220 mJ. SB+DL group was prepared using both techniques, just as described before.

The treated surface of all zirconia slabs was etched with 10% hydrofluoric acid for 60 seconds to remove remaining particles and greasiness, rinsed for 60 seconds, and air-dried. Then, one layer of one-bottle universal adhesive (Single Bond Universal, 3M ESPE, St. Paul, MN, USA) was actively applied on each treated surface for 20 seconds, gently air-dried, and light-cured for 10 seconds using a LED curing unit (Valo, Ultradent Products, South Jordan, UT, USA) with an output of at least 1000 mW/cm2, verified with a radiometer. On each zirconia slab, four plastic tubes (Kavo, Joinville, SC, Brazil) with an internal diameter of 0.8 mm and a height of 1 mm were positioned over the treated surface. Each tube was firmly held with a tweezer and carefully filled with the aid of a metallic probe with: 1) DUAL = equal amounts of base and catalyst paste of an amine-free dual-cure resin cement (Rely X Ultimate, 3M ESPE) mixed with the aid of a spatula for 10 seconds; or 2) LC = light-cure resin cement (Rely X Veneer, 3M ESPE) dispensed from a one-component syringe. The composite excesses were carefully removed, and the specimens were light-cured for 40 seconds using the LED curing unit (Valo, Ultradent).

-Michoshear Bond Strength Tests

After 48-hour storage in distilled water at 37°C, the tubes were carefully removed using a sharp blade, and the resin cement cylinders were exposed. Each zirconia slab was attached to a shear-testing jig in a universal testing machine with a 50 Kgf load cell (EZ test Shimadzu, Miyazaki, Japan). Then, each resin cement cylinder was looped by a 0.3-mm diameter metallic wire and the zirconia/resin cement interface was individually tested under shear mode at a crosshead speed of 0.5 mm/min until failure, and the µSBS was calculated in MPa. Two resin cement cylinders per slab were maintained and not tested. The slabs were stored again in distilled water at 37°C for 12 months and then, the two remaining cylinders were tested as described before.

-Failure Mode

The failure mode of each resin cement cylinder was determined using a 30x magnification stereomicroscope (EK3ST, Eikonal, São Paulo, SP, Brazil) and classified as adhesive, mixed, or cohesive.

-Statistical analysis

The µSBS data were statistically analyzed with a three-way analysis of variance (ANOVA), in which ‘surface treatment’ and ‘resin cement’ were assigned as main plots and ‘time’ was assigned as a split-plot. The multiple comparisons were performed using the Tukey Kramer test at a significance level of α=0.05 using a software package (R Core Team, R Foundation for Statistical Computing, Vienna, Austria). The association between resin cement and failure modes was analyzed by using Fisher’s exact test (*p*<0.05).

## Results

Only after 12 months of water storage, the µSBS of the DUAL resin cement was significantly higher than the LC resin cement in zirconia surfaces treated with SB or SB+DL (*p*<0.05). In addition, the surface treatment had a significant effect only for DUAL resin cement, which presented significantly higher µSBS when the zirconia was treated with SB+DL in comparison to DL (*p*<0.05). In comparison to the 48-h evaluation, the 12-month µSBS values of DUAL resin cement were significantly higher for zirconia surfaces treated with SB or SB+DL. The aging also had a significant effect on LC resin cement, which presented significantly higher 12-month µSBS values when the zirconia was treated with DL (*p*<0.05) ( Table 2).

**Figure 1:**
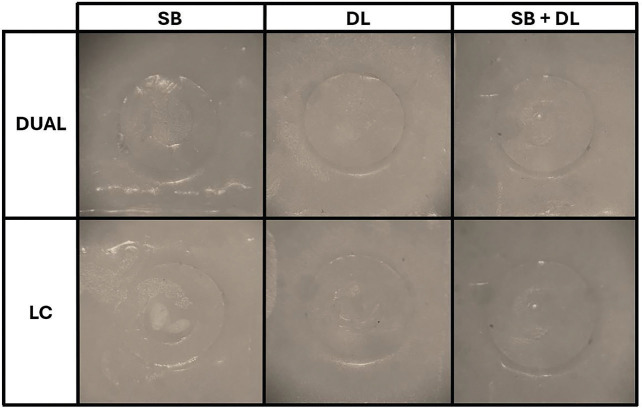
Representative images of the predominant failure mode of each group after 48 hours. The predominant failure mode for the samples luted with dual-cure resin cement was adhesive, for either SB-, DL- or SB+DL-treated zirconia. For samples luted with light-cure resin cement, the predominant failure mode was mixed for the SB-treated zirconia, and adhesive for DL- and SB+DL-treated specimens. Abbreviations: SB: sandblasting with 50-µm Al2O3, particles; DL: diode laser irradiation; DUAL = dual-cure resin cement; LC = light-cure resin cement.

A significant association between resin cements and failure modes was observed at both evaluation times (*p*<0.01) ( Table 3). At 48-h evaluation, most of the DUAL specimens presented adhesive failures. However, LC specimens luted on SB-treated zirconia presented 55% mixed failures and only 25% adhesive failures. Moreover, 40% of the LC specimens luted on SB+DL-treated zirconia showed mixed failures. Representative images of the predominant failure mode of each group after 48 hours are presented in Figure 1.

**Figure 2:**
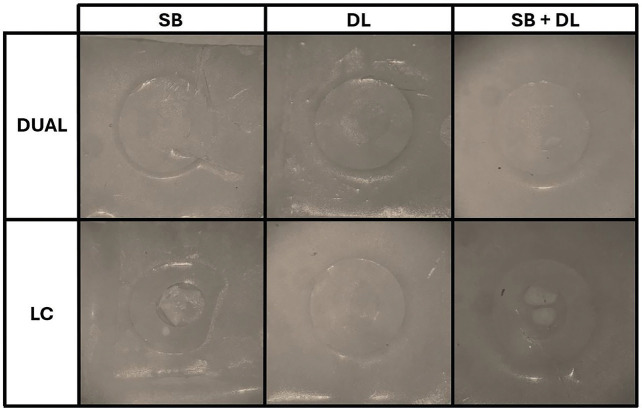
Representative images of the predominant failure mode of each group after 12 months. The predominant failure mode for the samples luted with dual-cure resin cement was adhesive, for either SB-, DL- or SB+DL-treated zirconia. For samples luted with light-cure resin cement, the predominant failure mode was mixed for the SB- and SB+DL-treated zirconia, and adhesive for DL-treated specimens. Abbreviations: SB: sandblasting with 50-µm Al2O3, particles; DL: diode laser irradiation; DUAL = dual-cure resin cement; LC = light-cure resin cement.

After 12 months, the majority of DUAL specimens luted on DL or SB+DL-treated zirconia presented adhesive failures. In addition, 35% of DUAL specimens showed mixed failures when luted on SB-treated zirconia. Most of the LC specimens luted on SB- or SB+DL-treated zirconia presented mixed failures, while DL-treated zirconia showed 50% adhesive and 45% mixed failures. Representative images of the predominant failure mode of each group after 12 months are presented in Figure 2.

## Discussion

Effective adhesive bonding to zirconia restorations relies on a combination of surface treatment and luting agent that provides strong and durable chemical bonds (8). Thus, this study evaluated the µSBS of different resin cements luted to high-translucent zirconia surfaces treated with sandblasting and/or laser irradiation. The results indicated that the null hypothesis was accepted after 48 hours and had to be rejected after 12 months.

Zirconia is known as an acid-resistant material due to its high metallic oxide content; thus, hydrofluoric acid etching has proven to be ineffective since it does not cause structural alterations on the surface of conventional or high-translucent zirconia (22). The surface treatment must provide adequate roughness without increasing opacity and light diffraction throughout the zirconia (20). Although Al2O3 sandblasting is highly effective, it can jeopardize the flexural strength of high-translucent zirconia with low yttrium content (23).

The use of laser irradiation for surface treatment of zirconia has already been reported in literature. The Er,Cr:YSGG laser can be used to treat zirconia without causing superficial damage (12). The Nd:YAG laser fuses the most superficial layer into a smooth and blister-like surface that increases the bond strength to resin cement (11). In contrast to glass-ceramic, the FEMTO laser (1030 nm) irradiated under different parameters resulted in microcracks that increased the surface roughness of high-translucent zirconia (6Y-PSZ), as well as enhanced its long-term mechanical properties (23).

Diode laser, in turn, had never before been investigated in this aspect. Although it does not produce surface damage of zirconia (18), diode laser used in a wavelength of 970 nm did not significantly differ from sandblasting for µSBS after 12 month-storage, regardless of the resin cement. However, when diode laser was associated with sandblasting, this combination resulted in higher µSBS for dual-cure resin cements after 12 months in comparison to the use of diode laser irradiation alone. The association of laser irradiation with sandblasting has already been reported in literature. Sandblasting with 50- or 90-µm Al2O3 particles and FEMTO laser irradiation showed similar effects on high-translucent zirconia surface, albeit sandblasting resulted in more irregular microcracks. However, higher shear bond strength values to resin cement were recorded for laser-irradiated zirconia surfaces (13).

In this study, the combination of sandblasting and diode laser (SB+DL) resulted in the highest µSBS values among the surface treatments; in addition, the 12-month µSBS values were significantly higher for light-cure resin cement luted on diode laser-treated zirconia. Although there is a lack of studies on diode laser irradiation, it can be expected that this surface treatment provides adequate long-term adhesion between zirconia and resin cement (24).

After 48-hour storage in distilled water, µSBS values were not significantly different regardless of surface treatment and resin cement type; thus, the null hypothesis was accepted. This may be explained by the similar composition of both resin cements and the standardized light-curing protocol. The polymerization reaction depends on the irradiation source, distance, and exposure time (21). Moreover, the physical properties such as the hardness of dual resin cements can increase after 24 hours due to a delayed increase in the polymeric chain (19). Garcia *et al*. (25) reported that dual-cure and light-cure resin cements luted on high-translucent zirconia showed similar shear bond strength after 24-hour distilled water storage.

The greater the thickness of the restorative material, the greater the intensity and exposure time of light curing. A low degree of monomer conversion jeopardizes the mechanical properties (shear strength, hardness, and modulus of elasticity), compromises the bonding interface due to increased water sorption and solubility, as well as causes optical changes in the resin cements (21). Although initiated by light irradiation, the polymerization reaction of dual-cure resin cements remains ongoing, which explains their hardness increase after 48 hours (19,26). Therefore, in this study, the dual-cure resin cement exhibited significantly higher µSBS after 12 months of distilled water storage than light-cure resin cement when luted on sandblasting- or SB+DL-treated zirconia; thus, the null hypothesis had to be rejected. Garcia *et al*. (25) also reported a significant increase in the shear bond strength between the same dual-cure resin cement luted on zirconia after 12 months of storage in distilled water. This indicates that aging allows the delayed increase of the polymeric chains and conversion degree of unreacted monomers. The increase of shear bond strength after aging suggests enhanced long-term resistance against hydrolytic degradation and mechanical failure.

Not only the surface treatment and the resin cement can influence the bond strength, but the adhesive system also plays a major role in this scenario. The one-bottle universal adhesive used in this study contains silane, which plays a key role in the chemical interaction between the luting agent and the inner surface of zirconia restoration, and has been associated with higher bond strength of resin cements (27). The presence of silica fillers on the luting interface tends to increase the modulus of elasticity and consequently enhances the mechanical strength (28). The silanization increases the surface wettability and thus enhances the bond strength between the silica groups of the restorative material and the methacrylate groups of the luting agent (29).

Even though the shear bond strength values found in the present study are similar to those found by other authors who also performed sandblasting as surface treatment of high-translucent zirconia (25,30), Dal Piva *et al*. (31) found considerably higher MPa values of resin cements luted to high-translucent zirconia. It can be hypothesized that this difference is related to the use of a separate single-bottled silane, which could be associated with higher shear strength values when compared to the use of a universal adhesive containing silane.

Most of the dual-cure resin cement specimens showed adhesive failures, while the majority of light-cure resin cement specimens exhibited mixed failures for sandblasting- or diode laser-treated zirconia. These findings are in agreement with other studies that also reported adequate bond strength between resin cement and zirconia, albeit not enough to withstand long-term hydrolytic degradation (30,32).

Even though light-cure resin cements may be preferred by clinicians when luting esthetic veneers due to their easy handling and controlled time to seat the veneers before initiation of the polymerization reaction (3), based on the findings of this study, it can be concluded that the dual-cure resin cement luted on sandblasting- or SB+DL-treated zirconia showed better long-term bond strength stability than light-cure resin cement *in vitro*. The light-cure resin cement luted on diode laser-treated zirconia showed increased µSBS after 12 months. Still, the decision making of which resin cement to use when luting high-translucent zirconia cannot be carried out based on an *in vitro* study. Our results inspire the conduction of future research aiming to investigate if this conclusion is reflected in a clinical level.

## Figures and Tables

**Table 1:** Materials used for adhesive luting.

Brand and manufaturer	Composition
Ceramill Zolid FX Multilayer 71M, AmannGirrbach, Koblach, Austria	ZrO2 + HfO2 + Y2O3 ≥99%; Y2O3: 6-7%; HfO2: ≤5%; Al2O3: ≤0.5%; other oxides ≤1%
Rely X Ultimate (A2 shade), 3M ESPE St Paul, MN, USA	55-65% silane-treated ceramic fillers, 10-20% TEGDMA 10-20% Bis-GMA, 1-10% silane-treated silica, <5% functionalized dimethacrylate polymer, <0.5% EDMAB, <0.5% benzotriazole), <0.5% diphenyliodonium hexafluorophosphate, <0.2% triphenylantimonium
Rely X Veneer (A2 shade), 3M ESPE, St Paul, MN, USA	55-65% silane-treated ceramic fillers, 10-20% TEGDMA 10-20% Bis-GMA, 1-10% silane-treated silica, <5% functionalized dimethacrylate polymer, and water
Single Bond Universal, 3M ESPE, St Paul, MN, USA	Bis-GMA, 2-hydroxyethyl methacrylate, silicon-treated silica, ethyl alcohol, decamethylene dimethacrylate, water, 1,10-decanediol phosphate methacrylate, copolymer of acrylic and itaconic acid, camphorquinone, N,N-dimethylbenzocaine, 2- methacrylate dimethylammonoethyl, methyl ethyl ketone
Porcelain conditioner, Dentsply, Petrópolis, RJ, Brazil	10% hydrofluoric acid, water, thickener and dye

Bis-GMA: bisphenol-A-diglycidylether dimethacrylate; TEGDMA: triethylene glycol dimethacrylate; EDMAB: 2-ethyl-4-aminobenzoate

**Table 2:** Mean µSBS (MPa±standard deviation) at different times in function of different surface treatments and resin cement types.

Time	Surface treatment	Resin cement	
		DUAL	LC
48 hours	SB	3.72 (±0.85)^Aa^	1.66 (±1.24)^Aa^
	DL	4.47 (±1.48)^Aa^	2.70 (±1.06)^Aa^
	SB + DL	5.17 (±0.93)^Aa^	3.25 (±1.77)^Aa^
12 months	SB	*7.71 (±2.74)^Aab^	4.44 (±2.50)^Ba^
	DL	5.69 (±2.40)^Ab^	*5.76 (±2.43)^Aa^
	SB + DL	*9.67 (±2,69)^Aa^	5.37 (±2.09)^Ba^

Abbreviations: SB: sandblasting with 50-µm Al2O3, particles; DL: diode laser irradiation; DUAL = dual-cure resin cement; LC = light-cure resin cement. Means with the same capital letters in the same row are not significantly different (*p*<0.05). Means with the same lowercase letters in the same column are not significantly different (<0.05). * Significantly different from 48-hour value at the same surface treatment and resin cement conditions (<0.05).

**Table 3:** Failure mode distribution.

Time	Resin cement	Surface treatment	Failure mode		
			Adhesive	Cohesive	Mixed
48 hours	DUAL	SB	12 (60%)	4 (20%)	4 (20%)
		DL	16 (80%)	0 (0%)	4 (20%)
		SB + DL	17 (85%)	0 (0%)	3 (15%)
	LC	SB	5 (25%)	4 (20%)	11 (55%)
		DL	15 (75%)	0 (0%)	5 (25%)
		SB + DL	11 (55%)	1 (5%)	8 (40%)
p-value			0.0011		
12 months	DUAL	SB	9 (45%)	4 (20%)	7 (35%)
		DL	14 (70%)	1 (5%)	5 (25%)
		SB + DL	16 (80%)	1 (5%)	3 (15%)
	LC	SB	2 (10%)	1 (5%)	17 (85%)
		DL	10 (50%)	1 (5%)	9 (45%)
		SB + DL	5 (25%)	2 (10%)	13 (65%)
p-value			<0.0001		

Abbreviations: SB: sandblasting with 50-µm Al2O3, particles; DL: diode laser irradiation; DUAL = dual-cure resin cement; LC = light-cure resin cement.

## Data Availability

The datasets used and/or analyzed during the current study are available from the corresponding author.
